# P-glycoprotein and Alzheimer’s Disease: Threats and Opportunities

**DOI:** 10.1080/17590914.2025.2495632

**Published:** 2025-04-23

**Authors:** Joseph Asante, Steven W. Barger

**Affiliations:** a Graduate Program in Bioinformatics, University of Arkansas at Little Rock, Little Rock, AR, USA; b Department of Geriatrics, University of Arkansas for Medical Sciences, Little Rock, AR, USA; c Department of Neuroscience, Little Rock, AR, USA; d Geriatric Research, Education & Clinical Center, Central Arkansas Veterans Healthcare System, Little Rock, AR, USA

**Keywords:** Alzheimer’s disease, P-glycoprotein, multi-drug resistance protein, ABC transporter, Amyloid β-peptide

## Abstract

Alzheimer’s disease (AD) is a progressive neurodegenerative disorder that affects more than 50 million people worldwide. One of the hallmark features of AD is the accumulation of amyloid β-peptide (Aβ) protein in the brain. P-glycoprotein (P-gp) is a membrane-bound protein expressed in various tissues, including the cerebrovascular endothelium. It plays a crucial role in the efflux of toxic substances, including Aβ, from the brain. Aberrations in P-gp levels or activity have been implicated in the pathogenesis of AD by promoting the accumulation of Aβ in the brain. Therefore, modulating the P-gp function represents a promising therapeutic strategy for treating AD. P-gp has multiple substrate binding sites, creating the potential for substrates to fall into complementation groups based on these sites; two substrates in the same complementation group may compete with one other, but two substrates in different groups may exhibit cooperativity. Thus, a given P-gp substrate may interfere with Aβ efflux whereas another may promote clearance. These threats and opportunities, as well as other aspects of P-gp relevance to AD, are discussed here.

## Introduction

Alzheimer’s disease (AD) is a complex neurodegenerative disorder that affects more than 50 million people worldwide. AD is characterized by progressive cognitive decline, memory loss, and changes in behavior and personality. It is the most common cause of dementia, accounting for 60–80% of all cases (“2023 Alzheimer’s Disease Facts and Figures,” 2023). The pathological hallmarks of AD include the accumulation of amyloid β-peptide (Aβ) plaques, neurofibrillary tangles (NFTs), and neuronal loss in the cerebral cortex and a few subcortical nuclei. Despite decades of research, the exact pathological progression of AD is still unclear (Hardy & Higgins, 1992).

Several aspects of AD pathogenesis involve alterations in the structure and function of the blood-brain barrier (BBB), a multifactorial phenomenon that effects maintenance of a specific and highly regulated environment in the CNS which is critical to its normal health and function (Huang et al., 2020; Langen et al., 2019; Zhao et al., 2015). The BBB has a physical component comprising cerebrovascular endothelial cells and the tight junctions formed between them by proteins such as claudin, occludin, and zonula occludens 1; together, these structures efficiently exclude water-soluble molecules from entering or leaving the CNS. However, lipophilic molecules are capable of diffusing through the membranes endothelial cells. Removal of these agents depends on a functional component of the BBB comprising active transport mechanisms, chief among them p-glycoprotein (P-gp) (Miller, 2015).

P-gp, a member of the ATP-binding cassette (ABC) transporter family, is expressed in several peripheral tissues, including the liver, kidneys, and intestines; it plays a crucial role in the efflux of xenobiotics and toxins from the cell (Schinkel & Jonker, 2003). In the brain, P-gp is mainly localized in the apical membrane of the cerebrovascular endothelium, where it transports substrates from the endothelial cytosol to blood, thus contributing to the functional component of the BBB. P-gp dysfunction—aberration in either either levels or activity—has been implicated in the pathogenesis of AD, as studies have shown that P-gp expression and function are reduced in the brains of AD patients compared to healthy controls (Vogelgesang et al., 2002). Moreover, P-gp dysfunction has been shown to impair Aβ clearance from the brain, thus contributing to the accumulation of Aβ plaques (Hartz et al., 2010). This and other evidence indicate that Aβ is one of the substrates that P-gp removes from the central nervous system (CNS). In addition, the impact of P-gp on drug delivery to the brain complicates the development of effective AD therapies, as many potential treatments are substrates for P-gp and are thereby transported out of the brain (Pardridge, 2020).

This review aims to provide an overview of the current understanding of P-gp’s role in AD. We will explore the dual nature of P-gp as both a challenge and an opportunity in the context of AD. By examining all aspects of P-gp biochemistry, we hope to shed light on new avenues for research and development of therapeutics for AD.

## Structure and Physiological Role of P-Glycoprotein

P-gp, also known as ABC sub-family B member 1 (ABCB1), is a 170-kDa transmembrane protein that plays a critical role in the efflux of xenobiotics and toxins from cells (Hoffmeyer et al., 2000). It also contributes to the BBB by transporting certain molecules from the cerebrovascular endothelial cells and into the blood. P-gp is encoded by the *ABCB1* gene, which is located on Chromosome 7 in humans (Hamidovic et al., 2010). The protein is expressed in several tissues throughout the body, including the liver, kidneys, intestines, and cerebrovascular endothelial cells (Cascorbi, 2011). The basic parts of the protein are the transmembrane domains (TMD) and the nucleotide-binding domains (NBD).

## Dynamic Structure of P-Glycoprotein

**Figure 1.**
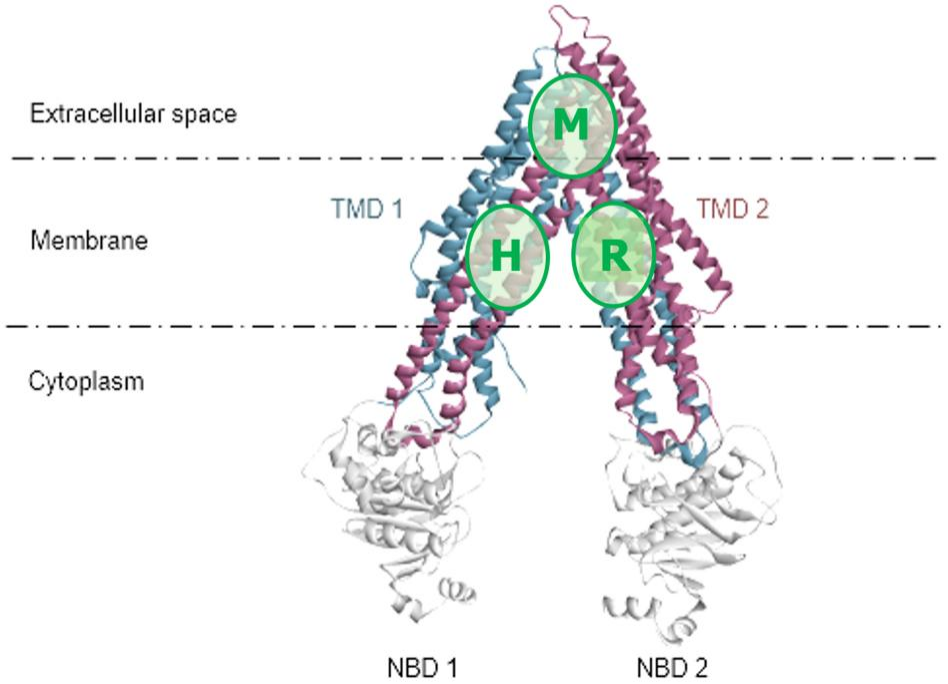
Representation of the inward-facing conformation of a mouse P-gp crystallographic structure (PDB ID: 4KSB). NBDs are in grey, and TMDs are in blue and magenta and with suggested drug-binding sites; H, R and M sites (Ferreira et al., 2017).

The structure of P-gp has been solved using X-ray crystallography and electron microscopy, revealing the detailed architecture of the protein in various combinations with nucleotides and transported substrates (Ward et al., 2013) (Figure 1). *In silico* molecular modeling has also been applied to predict the interaction of these structures in ways that might explain P-gp’s transport activity. These approaches have described a structure that is highly dynamic and adopts multiple conformational states during the transport cycle (Sauna & Ambudkar, 2007). These changes are thought to involve ATP-driven movements of the NBDs, which induce conformational changes in the TMDs that facilitate substrate binding, transport, and release. The precise mechanism by which P-gp transports substrates across the membrane is still not fully understood, but it is thought to involve a combination of conformational changes and substrate-induced movements within the TMDs (Sauna & Ambudkar, 2007).

P-gp consists of two halves that are homologous enough to create the appearance of a homodimer, though both halves are contained within a single, contiguous polypeptide; both the N-terminus and C-terminus are located on the cytoplasmic side of the membrane (Kim & Chen, 2018). Each of the halves has its own TMD comprising six membrane-spanning helices; each half also contains its own NBD (Ward et al., 2013). The two TMDs form a barrel-like structure that spans the lipid bilayer of the plasma membrane, creating a hydrophobic tunnel through which substrates can pass (Loo et al., 2003b). Each helix in a TMD contains 20–25 amino acid residues (Ward et al., 2013). The TMDs are responsible for substrate recognition and binding, as well as for the formation of the substrate translocation pathway (Sauna & Ambudkar, 2007). The NBDs are in the cytoplasmic domain of the protein and are responsible for binding and hydrolyzing ATP, which produces the energy for transport of substrates across the membrane (Choi, 2005).

P-gp contains at least two drug-binding sites (DBS) that have been identified in the TMDs through structural and biochemical studies: R and H (Biemans-Oldehinkel et al., 2006; Higgins, 2007; Ward et al., 2007). These have been suggested to interact in a positively cooperative manner, thus affecting substrate efflux and hence P-gp activity (Loo et al., 2003a; Shapiro & Ling, 1997). Substrates that share a binding site may competitively inhibit one another’s efflux (Shapiro & Ling, 1997). A third site (M) is located near the position where substrates would exit the enzyme at the apical promontory and appears to act as a more general modulator (Ferreira et al., 2017); binding at this site can inhibit the efflux of a substrate that binds either the R or H site. The substrate-binding sites are highly flexible and can adapt to different substrates, allowing P-gp to recognize and bind a wide range of structurally diverse molecules (Ward et al., 2013).

## Physiological Role of P-Glycoprotein

P-gp is a critical efflux transporter that plays a crucial role in protecting the body from toxic insults, pharmacokinetics, maintaining cellular homeostasis, and modulating immune responses (Gottesman et al., 2002). Its role in various physiological and pathological processes makes it an attractive target for the development of therapeutics.

1. Efflux of xenobiotics and toxins: P-gp is primarily known for its role in the efflux of a wide range of xenobiotics and toxins from cells, including drugs, environmental pollutants, and carcinogens. This function is particularly important in organs such as the liver, kidneys, and intestines, where P-gp is highly expressed (Cascorbi, 2011). P-gp transports these substances out of cells and into the extracellular space or blood, preventing their accumulation in tissues and organs, and facilitating their elimination from the body (Dunk et al., 2018; Lankas et al., 1998; Smit et al., 1999).
2. Protection of the brain: P-gp is also expressed in the luminal membrane of the endothelial cells that contribute to the BBB. Here, it plays a critical role in regulating the transport of substances across the barrier and protecting the brain from potential toxic insults (Sharom, 2011). The BBB is a selectively permeable barrier that separates the brain from the systemic circulation and regulates the transport of substances between the two compartments. One component of the BBB is physical: tight junctions between the cerebrovascular endothelial cells that block large polar and hydrophilic molecules from passing between blood and the brain interstitial fluid. But some lipophilic molecules pass directly through the endothelial cell membrane. Many of these are transported back to the blood by P-gp, reducing the entry of potentially harmful substances into the brain (Cascorbi, 2011; Janneh et al., 2005).
3. Roles in pharmacokinetics: Through its ability to redistribute xenobiotics, P-gp significantly impacts the pharmacokinetics of drugs. The efflux of drugs by P-gp can significantly affect their bioavailability, absorption, distribution, metabolism, and excretion (Lin & Yamazaki, 2003; Terasaki & Hosoya, 1999). The effects of P-gp in this regard are complex. P-gp can decrease the oral bioavailability of drugs by limiting their absorption from the gastrointestinal tract and increasing their efflux into the gut lumen (Miller et al., 2008), and its presence on the luminal brush-border membrane of renal epithelium can increase a drug’s clearance into the urine. However, the presence of P-gp on the luminal membrane of hepatocytes—facing the bile ducts—can reduce the time a drug spends in compartments containing cytochrome P450 enzymes (Lin & Yamazaki, 2003). P-gp can also limit the penetration of drugs into various tissues, including the brain, by effluxing them back into the blood or extracellular space (Kalvass et al., 2013). This function can have significant clinical implications, especially in the context of drug interactions, where P-gp inhibition or induction can alter the pharmacokinetics and efficacy of co-administered drugs (Chaudhary & Roninson, 1991).
4. Role in cellular homeostasis: P-gp also plays a role in maintaining cellular homeostasis by regulating the transport of endogenous substrates, such as lipids, steroids, and peptides (Sharom, 2008). P-gp can transport these substances out of cells, helping to maintain their concentration gradients and prevent their accumulation in cells, which could be detrimental to cellular function (Schinkel, 1999). This function is particularly important in organs such as the liver, where P-gp helps to regulate the excretion of bile acids and maintain cholesterol homeostasis (van Helvoort et al., 1996).
5. Immune modulation: P-gp expression has been detected in various immune cells, including T cells, B cells, and dendritic cells (Bossennec et al., 2018), and P-gp inhibition has been shown to modulate immune cell migration and function (Laupèze et al., 2001). Moreover, P-gp has been shown to transport cytokines such as interleukin-2 and interferon-γ, suggesting that it may play a role in the regulation of inflammatory responses (Wyska, 2009).
6. Role in cancer chemotherapy: P-gp overexpression is a common mechanism of resistance to chemotherapy in various types of cancer (Ambudkar et al., 2003; Szakács et al., 2006). The efflux of chemotherapeutic drugs by P-gp can limit their accumulation in cancer cells, reducing their cytotoxicity and efficacy. Therefore, P-gp inhibition has been proposed as a potential strategy to overcome drug resistance in cancer (Wu et al., 2011).

## The “Amyloid Hypothesis”

Aβ is a 39- to 43-amino-acid peptide that is normally produced in the body and abundantly in the brain (Hardy & Selkoe, 2002). It is derived from a larger protein called the amyloid precursor protein (APP) through the action of enzymes called secretases (Haass & Selkoe, 2007). A physiological role for Aβ is unclear, but its remarkable sequence conservation across 400 million years of evolution suggests that it has some important function(s); among those implicated are roles in synaptic plasticity and maintenance, regulation of cholesterol transport, and clearance of metal ions (Selkoe & Hardy, 2016).

Regardless of its ubiquitous production, Aβ production and/or accumulation becomes abnormal in certain conditions, such as AD (Citron, 2010). Aβ peptides are prone to aggregate and form insoluble deposits, which are believed to contribute to the neurodegeneration and cognitive impairment characteristic of AD (Uversky, 2009). The exact mechanism by which Aβ causes damage to brain cells is not fully understood (Dong et al., 2012), but it may involve a combination of oxidative stress, inflammation, and disruption of cellular signaling pathways (Chen et al., 2017). Comprising a small percentage of AD cases, familial AD (FAD) emanates from specific autosomal-dominant mutations which elevate the production of longer, more aggregative forms of Aβ; this explicates the causative role of Aβ accumulation. However, roughly 95 percent of cases are sporadic (i.e., of unknown etiology), and these appear to involve accumulation of Aβ without over-production, implying a problem with clearance of the peptide (Mawuenyega et al., 2010).

Several lines of evidence indicate that Aβ is involved in the pathogenesis of AD, either through the formation of senile plaques or through the elevation of soluble, diffusible multimers of the peptide. Whether there is a normal function of Aβ in the brain is still not fully understood, but several roles have been proposed. Aβ may play physiological roles in synaptic plasticity and neuronal development (Hardy & Selkoe, 2002; Walsh & Selkoe, 2007). However, excessive accumulation of Aβ can lead to neurotoxicity, oxidative stress, inflammation, and ultimately neuronal death, which are hallmarks of AD pathology (Bitan et al., 2003; Englander et al., 2007). Aβ can form a variety of different oligomeric and fibrillar structures, including soluble oligomers, protofibrils, and insoluble fibrils, which are known to be neurotoxic (Kuperstein et al., 2010; Mucke & Selkoe, 2012). Aβ can also bind to a variety of different receptors and molecules in the brain, including the acetylcholine receptor, N-methyl-D-aspartate (NMDA) receptor, α7 nicotinic receptor, prion protein, and glycosaminoglycans (Puzzo et al., 2008; Shankar et al., 2008). Aβ can modulate the activity of these receptors, leading to changes in synaptic plasticity and neuronal function. Overabundance of Aβ could create neurological problems simply by disrupting the balance of what are otherwise normal activities (*vis-à-vis* exerting entirely abnormal toxicity).

The “Amyloid Hypothesis,” i.e., the proposition that Aβ is important to AD pathogenesis, has been criticized extensively. It is clear, for instance, that some individuals can accumulate high concentrations of Aβ while retaining cognitive function within the normal range (Aizenstein et al., 2008). The converse is also true: dementia can occur with no clear evidence of Aβ accumulation above that seen in normal aging (Mormino & Papp, 2018). However, none of these facts is inconsistent with the hypothesis that Aβ is capable of initiating a chain of events that often results in dementia eventually. A role for Aβ is strongly suggested by the effects of FAD mutations, which all elevate the absolute or relative levels of Aβ_1-42/43_ (Citron et al., 1992). Timelines have also been established which place Aβ accumulation as the earliest common process in AD pathogenesis (Sperling et al., 2011). Individuals who accumulate considerable Aβ without dementia may simply have benefited from a break in the chain of events downstream of Aβ. This seems quite clear in the handful of cases wherein the carrier of a high-penetrance FAD mutation has been spared from dementia—as well as glucose aberrations, tau pathology, and cerebral atrophy—by a genetic variation in the apolipoprotein E gene (*APOE*) pathway, despite very high accumulation of Aβ (Raulin et al., 2022; Troutwine et al., 2022).

Given that tau pathology and other factors downstream of Aβ are likely required for the development of dementia, the impact of P-gp on these later events is worth considering. Key among these considerations is recent research indicating that P-gp is involved in inflammatory responses in the CNS, partly by influencing microglial activation. This is relevant in AD and tauopathies, where neuroinflammation often coincides with the formation of tau pathology. Reduction of P-gp activity elevates microglial activation (Brzozowska et al., 2017). Microglia participate in tau pathology by contributing to transcellular propagation of paired-helical filaments (Asai et al., 2015) and promoting tau hyperphosphorylation of tau through activation of p38-MAP kinase and Jun N-terminal kinase (Atzori et al., 2001; Li et al., 2003). Tau preformed fibrils can induce inflammation in microglia (Dutta et al., 2023), and the neuroinflammatory factors these cells produce can modulate P-gp (Théron et al., 2003; Yu et al., 2007). Understanding how P-gp intersects with tau-driven inflammation could thus reveal new therapeutic targets for managing neuroinflammatory responses in tauopathies (Huang et al., 2019).

## Clearance of Aβ

If the Amyloid Hypothesis is accepted, the steady-state removal of Aβ from the brain throughout life is a crucial mechanism in the prevention of the accumulation of Aβ plaques and the subsequent development of AD. There are several mechanisms involved in the clearance of Aβ, including enzymatic degradation, receptor-mediated transport, and efflux from brain to blood. The last two will be elaborated here, as they are relevant to the function of P-gp.

### Receptor-Mediated Endocytosis

Receptor-mediated transport appears to clear Aβ from the brain via the peptide’s binding to several specific receptors on the surface of cells, mediating the internalization of Aβ (Paresce et al., 1996). This form of transport is key to several hypotheses about clearance, including both P-gp-dependent and -independentndependPietrzik stent mechanisms. For instance, even enzymatic degradation of Aβ within microglia requires import of the peptide; while some of this may take the form of phagocytosis of large aggregates, receptor-associated endocytosis may predominate (Chung et al., 1999; Heckmann et al., 2019;; Paresce et al., 1996).

Lipoprotein receptors (LRPs) are a diverse family of receptors that play a role in lipid metabolism, cellular signaling, and the transport of various molecules across cell membranes. These receptors are essential for maintaining cellular homeostasis and regulating the distribution of lipoproteins and other key substances. In AD, LRPs are particularly significant due to their involvement in the transport and clearance of Aβ. Among the various LRPs, LRP1 and Very Low-Density Lipoprotein Receptor (VLDLR) are suggested for modulating Aβ levels and influencing the progression of AD (Deane et al., 2004, 2008).

LRP1 is a multifunctional receptor involved in various physiological processes, including lipid metabolism and cell signaling. It is expressed on endothelial cells, neurons, and astrocytes, where it facilitates the uptake of ligands such as lipoproteins and proteases. LRP1 appears to play a role in the transport of Aβ across the BBB by binding to Aβ and mediating its internalization into endothelial cells. Several studies indicate that LRP1 is required for transport of Aβ across the BBB (Deane et al., 2004; Shibata et al., 2000; Storck et al., 2016; Zlokovic et al., 2010). Due to its exclusive localization to the basolateral membrane of vascular endothelial cells (Farfán et al., 2013), LRP1 has been hypothesized to carry out the first step of Aβ efflux. Thus, while LRP-1 may be necessary for the uptake of Aβ into endothelial cells, it is not sufficient for transporting Aβ entirely across an endothelial layer (Nazer et al., 2008). Moreover, some evidence suggests that efflux of Aβ is entirely independent of LRP1 (Candela et al., 2015; Ito et al., 2010), and other findings suggest that transport of Aβ by LRP1 requires cotransport with well characterized ligands such as α2-macroglobulin (amada et al., 2008. Additionally, LRP1 may influence the expression and function of other critical BBB transporters, including P-gp, though the data are inconclusive. Storck et al. (2021) reported that conditional knockout of LRP1 in brain endothelial cells reduced the expression of P-gp, creating the possibility that reductions in Aβ efflux under those conditions actually reflected lower P-gp activity. However, this seems unlikely since the same investigators reported that a loss-of-function mutation in LRP1 elevated P-gp levels (Storck et al., 2018), in which case Aβ efflux was still diminished. Mice lacking endothelial LRP1 were also reported to suffer protease-mediated tight junction degradation, extravasation of immunoglobulin into the brain parenchyma, and compromised BBB function (Nikolakopoulou et al., 2021; Storck et al., 2021). Nevertheless, this finding conflicts with a prior finding that LRP1 removal did not significantly impact BBB function (Storck et al., 2016).

VLDLR is also involved in lipid metabolism and is structurally similar to LRP1, with multiple ligand-binding domains that facilitate interactions with various ligands. It contributes to the regulation of Aβ levels by mediating its transport across the BBB by promoting Aβ internalization into endothelial cells. Disruptions in VLDLR function can affect Aβ metabolism and clearance, leading to increased plaque formation and neuronal damage associated with AD (Deane et al., 2008).

### Efflux across the BBB

Efflux across the BBB is probably the primary mechanism responsible for clearance of Aβ from the brain (Cirrito et al., 2005). It has been estimated in transgenic mice that 40–60 percent of Aβ produced in the CNS is cleared through peripheral mechanisms (Qosa et al., 2014; Xiang et al., 2015), and Shibata et al. (2000) estimated that efflux across the BBB occurs at a rate nearly seven times that of bulk flow of interstitial fluid, which would logically relate to clearance via the CSF and glymphatic systems. Measurements of Aβ transport directly across the BBB in humans suggest that this route is responsible for at least 25 percent of the peptide’s clearance (Roberts et al., 2014).

The physical component of the BBB also restricts the outward diffusion of harmful substances that are generated within the CNS, such as Aβ. Thus, the efflux of Aβ across the BBB must be facilitated by several transporters, including P-gp—also known as multidrug resistance protein 1 (MDR1)—which is an ABC transporter that belongs to the superfamily of efflux pumps (Bendayan et al., 2006; Daneman & Prat, 2015). P-gp is expressed on the luminal side of the BBB (i.e., the apical surface of endothelial cells), where it functions as an efflux transporter for a variety of substrates, including Aβ (Cirrito et al., 2005; Hartz et al., 2010; Wang et al., 2016). P-gp is capable of recognizing and binding to Aβ, and it actively transports Aβ out of the brain and into the bloodstream, thereby helping in the balance of the levels of Aβ in the brain (Van Assema et al., 2012; Wang et al., 2016).

Several studies have shown that P-gp expression and function are reduced in the brains of AD patients compared to healthy controls, which may contribute to the accumulation of Aβ in the brain (Ding et al., 2021; Wang et al., 2016). In addition, animal models of AD have shown that increasing P-gp expression or function can reduce Aβ levels in the brain and improve cognitive function (Mohamed et al., 2016). Therefore, P-gp is considered a promising target for the development of AD therapeutics that can enhance the efflux of Aβ across the BBB.

Other factors can influence the efflux of Aβ across the BBB, including inflammation, oxidative stress, and age-related changes in the BBB (Hartz et al., 2010; Tamagno et al., 2021). Chronic inflammation and oxidative stress can damage the BBB and impair the function of transporters, leading to a reduced efflux of Aβ from the brain (Zenaro et al., 2017). Also, Aβ is transported from the brain interstitial fluid into the cerebrospinal fluid (CSF) through diffusion, bulk flow, and specific transporters. Once in the CSF, Aβ is cleared via absorption into the bloodstream or drainage into the cervical lymph nodes through meningeal lymphatics (Da Mesquita et al., 2018; Roberts et al., 2014).

In summary, some evidence suggests that the clearance of Aβ across the BBB involves coordinated work between LRP1 and P-gp to remove Aβ from the brain. LRP1, present on the basolateral membrane of cerebrovascular endothelial cells, may bind to Aβ in the brain interstitial fluid and effect transcytosis of Aβ from the brain interstitial fluid into endothelial cells. P-gp, which is found exclusively on the apical membrane, would then execute the transcytosis of Aβ into the circulating blood, i.e. the luminal compartment (Hartz et al., 2010; Nazer et al., 2008; Storck et al., 2018). It is also worth mentioning that the late-onset AD risk factor phosphatidylinositol binding clathrin assembly protein (PICALM) has been identified as a functional, and perhaps physical, link between LRP1 and P-gp, guiding both proteins through the endocytotic pathway in cerebrovascular endothelium (Storck et al., 2018).

## P-gp and AD

As mentioned previously, P-gp is a key player in the BBB and has been shown to actively efflux a broad range of endogenous and exogenous substances, including Aβ peptides. Thus, alterations in P-gp expression or function may contribute to the pathogenesis of AD by allowing Aβ to accumulate in the brain. Indeed, several studies have demonstrated that P-gp expression and function are decreased in AD patients (Chiu et al., 2015; Vogelgesang et al., 2002, 2004; Wijesuriya et al., 2010).

### Interaction of Conventional AD Drugs and P-gp

Cholinesterase inhibitors (donepezil, galantamine, and rivastigmine) and an N-methyl-D-aspartate receptor antagonist (memantine) have been used in AD treatment for some years, with results varying between individual patients (Cummings et al., 2019). P-gp impacts their bioavailability and therapeutic efficacy variably, based on each drug’s structure and resulting affinity for P-gp. Among cholinesterase inhibitors, donepezil’s CNS concentration is most strongly impacted by P-gp activity (Spieler et al., 2020). Notably, differences in transporter expression between individual patients may contribute to the relative efficacy of the cholinesterase inhibitors across patients (Mahringer & Fricker, 2016). This can have a cascading effect on treatment options, as memantine is advised only as an adjuct for those on stable donepezil therapy (Deardorff & Grossberg, 2016).

### Impact of Reduced and Impaired P-gp Function in AD

Evidence indicates that P-gp function is compromised in AD as several factors contribute to this reduced activity, including aging, genetic variations, and pathological conditions associated with AD. For instance, P-gp expression was found to be significantly decreased in the cerebral cortex and hippocampus of AD patients compared to controls (Vogelgesang et al., 2004). Isolated brain capillaries from AD patients also show a reduction in P-gp levels (Jeynes & Provias, 2011). Measures of P-gp activity with positron emission tomography (PET) imaging of [^11^C]-verapamil also documented a diminution in AD (Deo et al., 2014).

There are several ways in which decreased P-gp function could contribute to AD pathogenesis, but it is likely that it would contribute to accumulation of Aβ in the brain, as studies have shown that P-gp can directly interact with Aβ and that blocking P-gp function decreases transport of Aβ (Kuhnke et al., 2007; Lam et al., 2001; Van Assema et al., 2012). In addition, P-gp knockout mice have been shown to have increased brain Aβ levels compared to wild-type mice (Hartz et al., 2010; Wang et al., 2016). Thus, decreased P-gp function may lead to increased brain Aβ levels, which in turn can lead to the formation of Aβ plaques and neuronal damage.

In addition to its role in Aβ efflux, P-gp has been implicated in the efflux of other molecules that may be relevant to AD pathogenesis, including inflammation-related factors. For example, P-gp has been shown to efflux pro-inflammatory cytokines and chemokines from brain endothelial cells, which may be important in modulating the inflammatory response in AD (Van Assema et al., 2012).

### Evidence of P-gp-Mediated Export of Aβ

*In vitro* studies have been crucial in understanding how P-gp mediates the export of Aβ and identifying potential therapeutic targets for AD. These studies utilize various experimental models, including primary cultured cells, immortalized cell lines, and artificial membrane systems to explore the molecular and cellular mechanisms of Aβ transport and P-gp function. For instance, comparative studies using non-neuronal CHO-APP cells and human neuroblastoma SK-N-SH cells have shown that P-gp is both expressed and active in these cell types (Chai et al., 2020). When P-gp activity was inhibited with verapamil and nicardipine, the secretion of Aβ was significantly impaired, highlighting P-gp’s critical role in Aβ export. This suggests that enhancing P-gp function could help remove excess Aβ from the brain, offering a potential therapeutic approach for AD.

Further characterization of P-gp’s interaction with Aβ was conducted using MDR1-transfected LLC cells in a polarized cell-layer model (Kuhnke et al., 2007). This model allowed researchers to monitor P-gp-mediated transport by tracking the efflux of the fluorescent dye rhodamine-123 and Aβ peptides into the apical extracellular space. The study demonstrated that Aβ significantly decreased the apical efflux of rhodamine-123 while confirming the transcellular transport of Aβ_1-40_ and Aβ_1-42_ into the apical chamber using ELISA and fluorescence-labeled peptides.

Correlational studies in human tissues also support the inverse relationship between P-gp expression and Aβ accumulation. Immunohistochemical analysis of brain tissue samples from 243 non-demented individuals aged 50 to 91 years revealed a significant inverse correlation between vascular P-gp expression and the deposition of Aβ_1-40_ and Aβ_1-42_ in the medial temporal lobe (Lam et al., 2001). These results suggest that P-gp may influence Aβ elimination, impacting its accumulation and the progression of AD.

In animal studies, P-gp’s role in Aβ transport across the BBB was investigated using two approaches. First, mice genetically ablated for *Mdr1a* or *Mdr1a* and -*b* were crossed with Tg2576 APP transgenic mice, known for Aβ accumulation, and Aβ levels in brain extracts were measured, either acutely, through microdialysis (Cirrito et al., 2005) or chronically using ELISA (Wang et al., 2016). In a second approach wild-type and *Mdr1*-knockout mice were compared following intracerebral (Cirrito et al., 2005) or intravenous (Wang et al., 2016) injection of [^125^I]-labeled Aβ (Wang et al., 2016). The results of all these experiments showed greater CNS Aβ accumulation in mice deficient for either *Mdr1a* or both murine P-gp genes. Another study provided indirect evidence by demonstrating that inhibiting P-gp degradation by proteasomes reduced Aβ levels in Tg2576 mice (Vulin et al., 2023). *In vivo* assays using loperamide, a P-gp substrate, demonstrated that P-gp function at the BBB was impaired in Tg2576 mice compared to wild-type controls, indicated by increased CNS penetration and subsequent antinociceptive effects of loperamide. A two-week treatment of Tg2576 mice with the proteasome inhibitor bortezomib (BTZ) restored P-gp levels to those in wild-type and returned the behavioral effects of loperamide to normal, suggesting that the BTZ elevated P-gp-mediated efflux of Aβ. The latter was reinforced by reductions in brain levels of Aβ_1-40_ and Aβ_1-42_ in BTZ-treated Tg2576 mice. Thus, this study went beyond empirical demonstrations of Aβ clearance by P-gp to provide a potential therapeutic approach to alleviate AD pathology.

Human studies have been compelling as well. One study compared BBB P-gp function in AD patients and age-matched healthy controls using (R)-[^11^C]verapamil and PET imaging (Van Assema et al., 2012). AD patients showed increased brain (R)-[^11^C]verapamil binding potential values, indicating decreased P-gp function, especially in the frontal, parietal, temporal, occipital cortices, and cingulate regions. These findings suggest altered (R)-[^11^C]verapamil kinetics in AD, reflecting decreased P-gp activity, similar to when P-gp is pharmacologically inhibited.

Finally, *in silico* studies using molecular modeling and simulation have predicted the interaction of P-gp with its substrates, including the identification of at least three druggable sites on P-gp (Ferreira et al., 2012, 2017; Prajapati et al., 2013; Prajapati & Sangamwar, 2014). One study predicted avid binding of Aβ_1-40_ and Aβ_1-42_ to P-gp (Callaghan et al., 2020), while another modeled the transport of Aβ_1-40_ and Aβ_1-42_ monomers by P-gp in simulations of the putative catalytic cycle. The movement of Aβ peptides by P-gp ranged from 8 Å to 20 Å across all simulations, providing insights into how P-gp might transport Aβ (McCormick et al., 2021).

### Threats: Challenges Posed by P-Glycoprotein in Alzheimer’s Disease

P-gp plays a dual role in AD, presenting both challenges and opportunities for treatment. While its protective function at the BBB is crucial for maintaining brain health, P-gp also poses some challenges that hinder the effective management of AD.

1. Broad substrate specificity: P-gp facilitates the removal of a wide variety of foreign and natural compounds from cells by using ATP hydrolysis for energy (Ambudkar et al., 2003; Sauna et al., 2007; Ueda et al., 1987). Its substrates include Aβ and other predominantly hydrophobic and mildly amphipathic substances, such as antibiotics, steroid hormones, chemotherapeutic agents, immunosuppressants, and anti-HIV protease inhibitors (Bikadi et al., 2011; Kim et al., 1998; Lee et al., 1998). Some of these molecules might inhibit the efflux of Aβ on binding to P-gp.
2. Multiple substrate binding sites: P-gp has been suggested to have at least two binding sites for transporting xenobiotics (Ferreira et al., 2017; Shapiro & Ling, 1997). Substrates that share a binding site (either at the H or R sites) may competitively inhibit one another’s efflux via P-gp (Amin, 2013). The M-site appears to act as a more general modulator (Ferreira et al., 2017), where binding can inhibit the efflux of substrates, including Aβ that may bind other sites. Verapamil, a well-established P-gp modulator, is predicted to bind to the M-site (Ferreira et al., 2017), suggesting that the effect of this agent on efflux of P-gp substrates could only be inhibitory.
  It is worth noting that computational studies of P-gp substrates often examine either a drug or Aβ and not P-gp’s binding and transport of a drug and Aβ, simultaneously. P-gp undergoes conformational changes upon substrate binding, which are critical to its transport function (Mollazadeh et al., 2018; Waghray & Zhang, 2018). The binding of one substrate may influence P-gp’s affinity and conformational state, potentially altering its ability for other substrates to bind and be transported. Thus, without evaluating these interactions together, studies may miss important dynamics in how P-gp handles Aβ clearance when a drug is also present.
3. Although P-gp deletion has not been observed in humans, subtler genetic variations that modulate P-gp activity or expression could have implications for AD. Certain P-gp single nucleotide polymorphisms (SNPs), like the exon 26 3435T allele, have been determined to reduce P-gp expression, leading to functional variations in the oral absorption and disposition of drugs. It is plausible that similar polymorphisms in P-gp may hinder Aβ transport out of the brain, thereby increasing the likelihood of plaque formation. Conversely, polymorphisms that enhance P-gp expression or transport activity might be expected to lower the risk of Aβ deposition and the subsequent development of AD. Nevertheless, some SNPs are likely to have effects on the binding of individual substrates rather than on the general rate of transport activity. More than 77,000 SNPs have been identified in the human ABCB1 locus, and determining the effects of even a fraction of these will be a difficult task, as evinced by the conflicting reports for those that have been studied in depth (Hoffmeyer et al., 2000; Marzolini et al., 2004; Wolking et al., 2015).

### Opportunities: Therapeutic and Research Potential

Despite the structural challenges posed by P-gp in AD, there are significant opportunities for therapeutic intervention and research advancements. Understanding and modulating P-gp’s function could pave the way for novel treatment strategies and enhance our comprehension of AD pathogenesis.

#### Therapeutic Potential

1. Enhancing P-gp levels: Because P-gp contributes to removing Aβ from the brain, enhancing its levels could reduce Aβ accumulation, a hallmark of AD. Treatment with a P-gp inducer such as St. John’s wort has been shown to increase the expression and function of P-gp in AD transgenic mice (Brenn et al., 2014). Drug therapies aimed at upregulating P-gp expression or activity could therefore be a novel approach to slowing or preventing AD progression (Teleanu et al., 2022)​.
2. Enhancing P-gp function: Strategies that boost P-gp activity independent of expression levels may improve Aβ clearance from the brain without affecting other important functions. Two substrates that interact with the two different binding sites on the transporter sometimes show cooperativity. Defining the Aβ binding site could foster exploration of agents that bind the complementary site to test whether any can boost Aβ efflux rates.
3. Combination therapies: P-gp modulators could be used alongside other AD treatments to improve drug delivery to the brain or to reduce the efflux of therapeutic agents that are substrates of P-gp, thus increasing their efficacy. For example, combining P-gp inhibitors with anti-amyloid drugs might enhance the drugs’ retention in the brain, potentially improving their effectiveness (Pardridge, 2020)​.

#### Research Potential

1. Biomarkers and diagnostic tools: Developing reliable biomarkers for P-gp function is crucial for advancing personalized medicine in AD. Non-invasive imaging techniques, such as PET with radiolabeled P-gp substrates, are being investigated to assess P-gp activity *in vivo*. These tools could help identify individuals with impaired P-gp function, allowing for tailored therapeutic interventions. Biomarkers that reflect P-gp activity could also be used to monitor treatment efficacy and disease progression (García-varela et al., 2021). By tracking changes in P-gp function, clinicians could adjust therapeutic strategies in real-time, optimizing patient outcomes.
2. Target for drug development: Identifying molecules that specifically modulate P-gp’s activity could lead to the development of new drugs aimed at reducing Aβ accumulation in the brain. High-throughput screening of compounds for P-gp interaction is an active area of research with potential to yield new therapeutic candidates ​(Pardridge, 2020).
3. Understanding AD pathogenesis: Research into the genetic and environmental factors that influence P-gp function can provide deeper insights into AD pathogenesis. For instance, studying how P-gp expression changes with aging or in response to chronic disease states can offer clues to the onset and progression of AD ​(Teleanu et al., 2022).

## Conclusion

**Figure 2.**
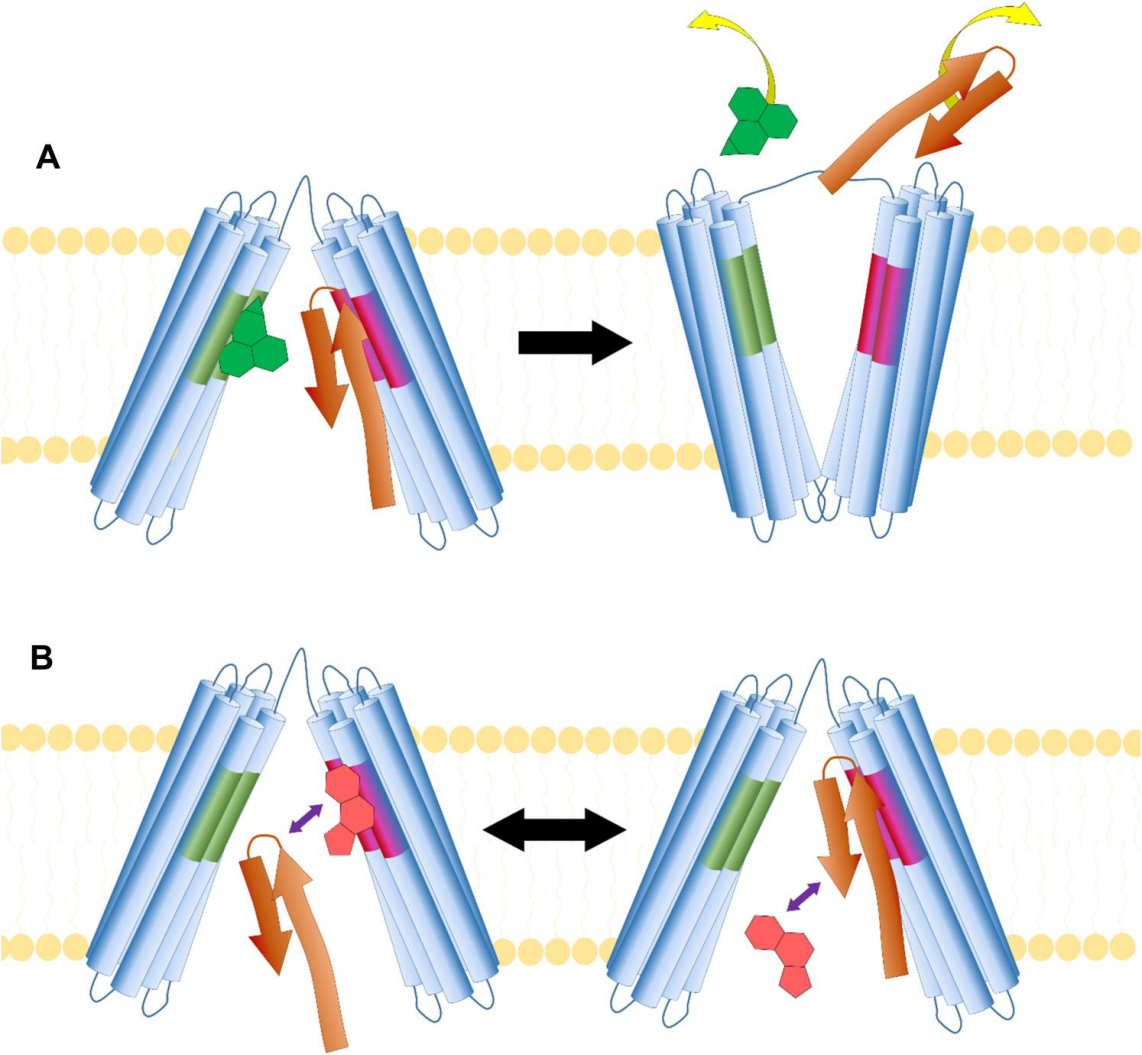
Hypothesis for cooperative versus competitive substrate interactions at P-gp. **Panel A:** A drug (green) and Aβ (rust) bind to different sites in the P-gp TMDs (blue; NBDs are omitted for simplicity), which hypothetically facilitates the efflux of each substrate. This could reduce the rate of Aβ accumulation in the CNS. **Panel B:** A drug (red) and Aβ (rust) compete for the same site on P-gp, likely to slow the efflux of each substrate. This could exacerbate Aβ accumulation in the CNS.

P-gp clearly represents a factor in the pathogenesis and treatment of AD. While its primary function at the BBB is to protect the brain by effluxing toxic substances, some molecules and other factors may pose a challenge to its activity. Approved medications are among the molecules that may inhibit the efflux of Aβ by competing for a binding site, and failure to appreciate this relationship could unintentionally promote accumulation of Aβ in the brain (Figure 2B). Conversely, it is possible that some drugs and other xenobiotics will enhance Aβ clearance by binding to a site distinct from that bound by Aβ (Figure 2A). The latter scenario might be exploited to identify molecules that are otherwise relatively inert and yet promote Aβ clearance. Regardless, of the practical feasibility of utilizing this potential therapeutic approach, it is clear that P-gp should be taken into consideration regarding any attempts to alleviate amyloid accumulation. A large body of research indicates that P-gp plays an essential role in removing Aβ from the CNS. Therefore, any condition that compromises its expression or function is likely to impact the risk of developing AD. This includes natural influences, such as genetic polymorphisms or xenobiotics in the diet, and iatrogenic factors, whether intended to affect Aβ clearance or not.

Addressing Aβ interactions with drugs that are substrates of P-gp may afford the potential to reduce Aβ accumulation and thereby reduce the incidence of AD as the impaired function of P-gp in AD patients increases Aβ accumulation, contributing to the progression of neurodegenerative processes.

## Data Availability

There is no original data associated with this publication.
